# Supplementary material for: Resolving the full spectrum of human genome variation using Linked-Reads
Source: Genome Res. 2019 Apr;29(4):635–45. doi: 10.1101/gr.234443.118 (PMC6442396; doi:10.1101/gr.234443.118)
Supplement: Supplemental Material [file supp_gr.234443.118_Supplemental_longranger-2.2.zip › longranger-2.2.tar.bz2!/lr2.2-public/lib/go/src/code.google.com/vitess/web/vtctld2/src/app/status/tablet-popup.component.html]

### Nothing selected. Hover over a point to show details.

### Tablet does not exist.

**{{title}}**

Keyspace: {{keyspace}}

Shard: {{shard}}

Cell: {{cell}}

Tablet type: {{tabletType}}

### Click on tablet for more details

**Aggregated tablets of all types**
  

Value: {{data}}

{{hostname}}

Go to tablet

|  |  |
| --- | --- |
| Replication Lag {{lag}} | |
| Qps {{qps}} | |
| Serving {{serving}} | |
| Health Error {{error}} | |
| Last Error {{lastError}} | |
